# Experimental data of four-point probe, scanning electron microscopy, and near-edge X-ray fine structure of titanium (IV) isopropoxide and zirconium (IV) dioxide binders incorporated carbon-based counter electrode for dye-sensitized solar cells

**DOI:** 10.1016/j.dib.2021.107487

**Published:** 2021-10-17

**Authors:** Nurnajaa Narudin, Piyasiri Ekanayake, Ying Woan Soon, Hideki Nakajima

**Affiliations:** a Faculty of Science, Universiti Brunei Darussalam, Jalan Tungku Link, Gadong BE 1410, Brunei Darussalam; b Optoelectronic Devices Research Group, Universiti Brunei Darussalam, Jalan Tungku Link, Gading BE 1410, Brunei Darussalam; c Synchrotron Light Research Institute, 111 University Avenue, Muang District, Nakhon Ratchasima 30000, Thailand

**Keywords:** Counter electrodes, Carbon black-graphite composite, Binders, Near-edge X-ray absorption fine structure, Four-point probe resistivity

## Abstract

In this article, we present the data collected from the fabricated carbon black-graphite counter electrode for dye-sensitized solar cells (DSSC) by incorporating binders such as titanium (IV) isopropoxide (TTIP), and zirconium (IV) dioxide (ZrO_2_). The addition of binders to the carbon black-graphite composite (CB/Gr) can drastically improve the adherence between the counter electrodes and the fluorine-doped tin oxide (FTO) substrate, surface area and the interparticle connection between the carbon materials. These data are presented which comprise of the resistivity measurements, scanning electron microscopy (SEM) with energy dispersive X-ray (EDX), and near-edge X-ray absorption fine structure (NEXAFS). The collection of this data was performed at room temperature. Detailed analysis of the data can be found in [1].

## Specifications Table

**Table:** 

Subject	Solar photovoltaics
Specific subject area	Carbon-based counter electrodes for dye-sensitized solar cells
Type of data	Table, Image, Figure
How data were acquired	4PP Model DFP-02, JEOL JSM-7610 FEGSEM, and CLAM 2, Thermo VG Scientific
Data format	Raw and analyzed (Processed)
Parameters for data collection	Varying amount of TTIP or ZrO_2_ binders added while keeping a constant ratio of carbon black:graphite counter electrode.
Description of data collection	The needles of the four-point probe (4PP Model DFP-02) are placed on the counter electrode film. Then, the current is set at a desired level and the voltage is measured. The SEM (JEOL JSM-7610 FEGSEM) utilized back scattered electron. The NEXAFS (CLAM 2, Thermo VG Scientific) is carried out at an angle of 20° from the surface normal.
Data source location	Institutions: 4PP Model DFP-02 and JEOL JSM-7610 FEGSEM are located at Universiti Brunei Darussalam, Brunei Darussalam. CLAM 2, Thermo VG Scientific is located at BL3.2Ua of the Synchrotron Light Research Institute, Thailand.
Data accessibility	Repository name: Mendeley Data Data identification number (DOI): 10.17632/xv3vs3hsxs.2 Direct URL to data: https://data.mendeley.com//datasets/xv3vs3hsxs/2[2] Instructions for accessing these data: open access
Related research article	Narudin, N., Ekanayake, P., Soon, Y. W., Nakajima, H., & Lim, C. M. (2021). Enhanced properties of low-cost Carbon Black-graphite counter electrode in DSSC by Incorporating binders. *Solar Energy, 225*, 237-244. https://doi.org/10.1016/j.solener.2021.06.070

## Value of the Data

• This data provides resistivity, SEM, and NEXAFS that can be used to investigate the effect of binders on low-cost CB/Gr counter electrodes on the surface adherence, conductivity and electrochemical activity for DSSC.
• Research on carbon-based counter electrodes can benefit from these data for experimental and validation which led to good adherence, high conductivity, high electrochemical activity, and low-cost counter electrodes for DSSC. It was imperative that binders provide good bonding among the carbon materials in the counter electrode and also between the counter electrode and FTO substrate interface to obtain improved photovoltaic performance of the device.
• Research on the addition of TTIP to CB/Gr counter electrodes that subsequently forms titanium dioxide (TiO_2_) in situ will give the potential for future work. The NEXAFS data from this article offer the possibility to engineer and optimize the energy levels of the counter electrode in order to acquire a high-performing DSSC.
• Meanwhile, conductivity data indicates the bonding between the carbon black and graphite in counter electrodes which is relatively influenced by the properties of the binders.
• Different binders with different particle sizes are utilized as the particle size of the binders can affect the performance of the counter electrodes. In this work, the particle size of TiO_2_ is measured using SEM.
• Identical data collection procedure was followed on all the fabricated counter electrodes; to ensure fair comparison of results from each binder.

## 1 Data Description

There are three levels of data included in this paper. The first level is the resistivity measurements of CB/Gr counter electrodes with different amount of TTIP and; 5 µm and 0.22 µm particle sizes of ZrO_2_ binders added. The resistivity and conductivity of the fabricated counter electrodes are summarized in Table 1.

**Table 1:** Resistivity and conductivity of the fabricated counter electrodes.

		Weight of	Thickness	Sheet resistance	Resistivity	Conductivity
	Counter electrodes	binders (g)	(µm)	(Ω Sq^−1^)	(x 10^−5^ Ω m)	(Sm^−1^)
(a)	CB/Gr:TTIP	0.010	12.0	5.09	6.11	16,367
		0.050	12.4	4.73	5.86	17,065
		0.075	12.2	4.69	5.72	17,483
		0.100	12.7	3.60	4.58	21,834
		0.150	12.1	3.74	4.52	22,124
(b)	CB/Gr:ZrO_2_ (5 µm)	0.010	12.5	7.77	9.71	10,299
		0.050	12.8	7.41	9.49	10,537
		0.075	12.2	7.09	8.66	11,548
		0.100	12.3	6.89	8.48	11,792
		0.150	12.1	6.82	8.25	12,121
(c)	CB/Gr:ZrO_2_ (0.22 µm)	0.010	12.3	5.92	7.28	13,736
		0.050	12.2	5.89	7.19	13,908
		0.075	12.9	5.44	7.02	14,245
		0.100	12.3	5.54	6.81	14,684
		0.150	12.1	5.49	6.64	15,060

**Fig. 1:**
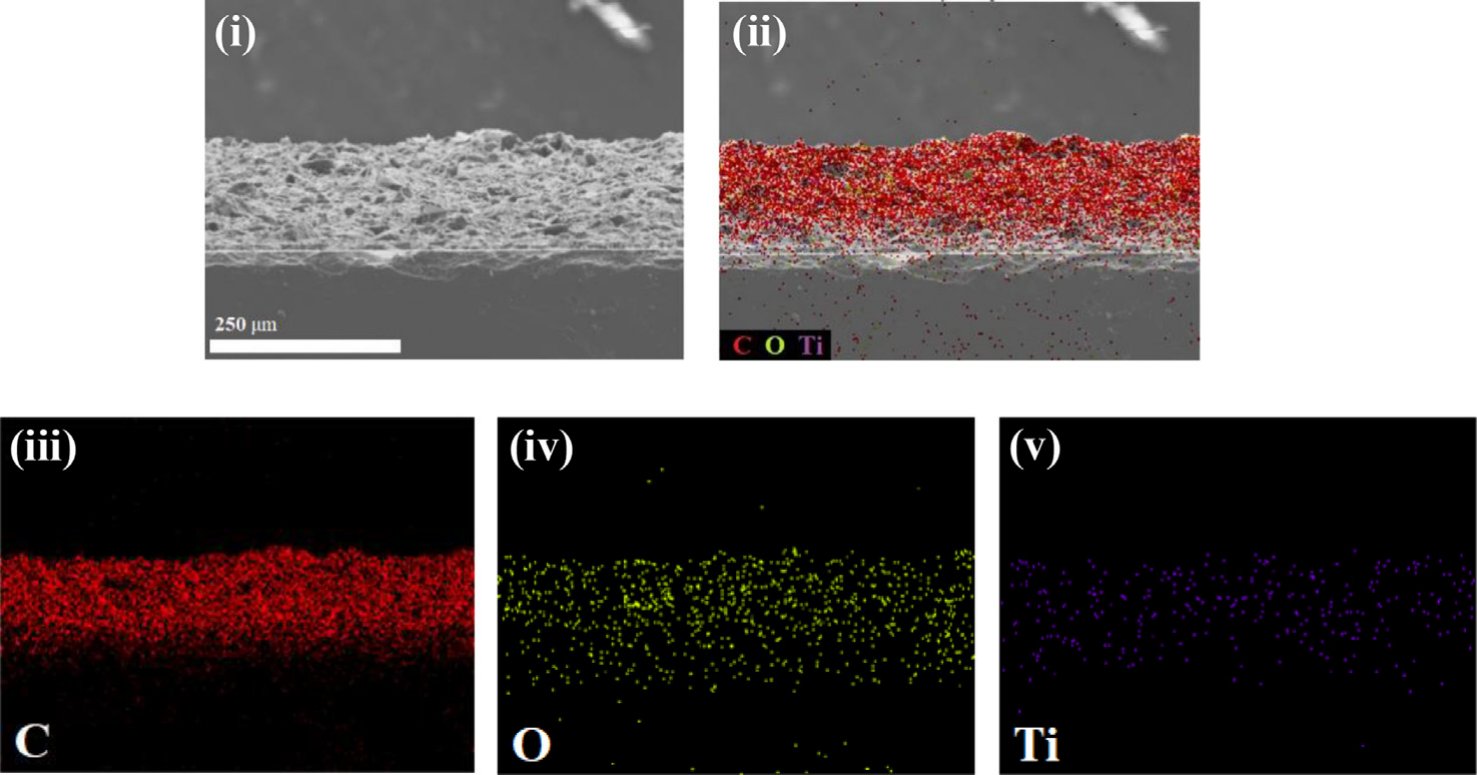
Side-view SEM-EDX images of CB/Gr:TTIP counter electrode.

**Fig. 2:**
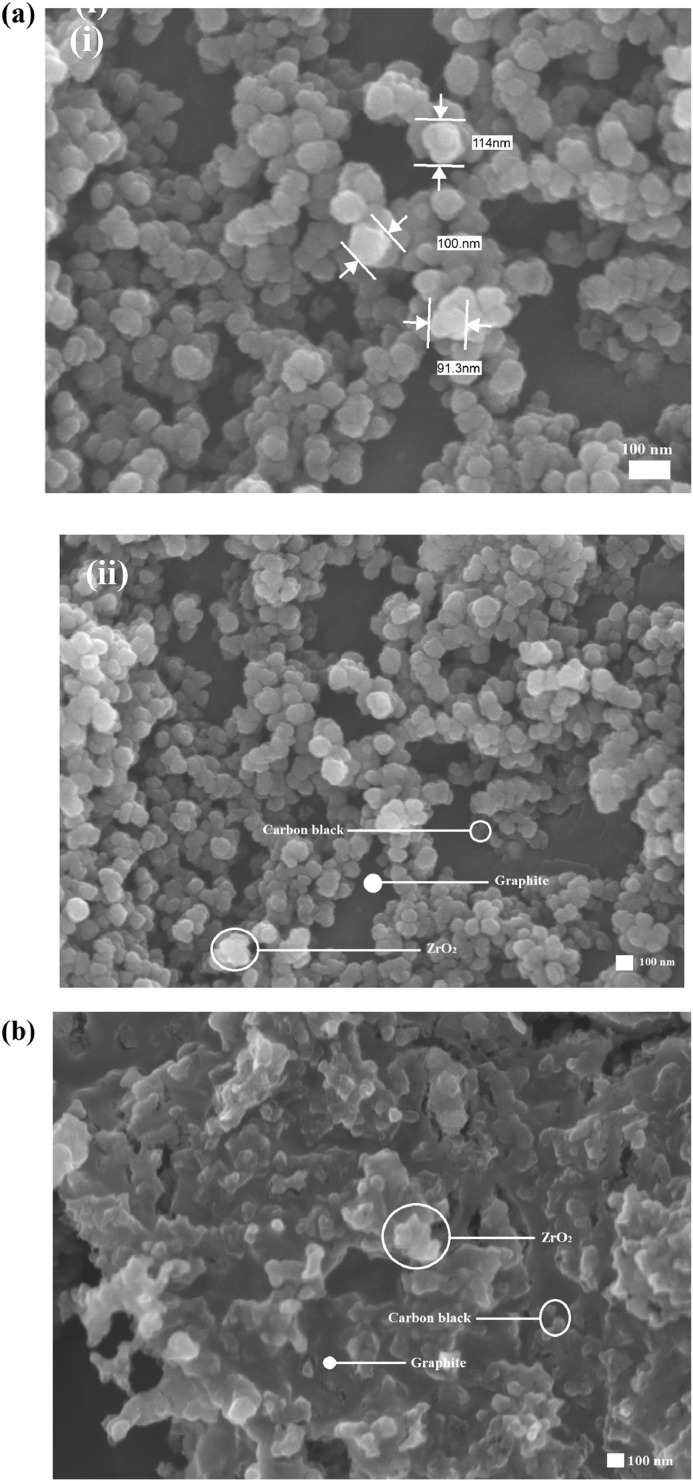
Top-view SEM images of **(a)** CB/Gr:ZrO_2_ (0.22 µm) and **(b)** CB/Gr:ZrO_2_ (5 µm).

The second level provides scanning electron microscopy with energy dispersive X-ray (SEM-EDX) images of side-view of CB/Gr:TTIP (see Fig. 1), and SEM images of top-view of CB/Gr:ZrO_2_ (0.22 µm) and CB/Gr:ZrO_2_ (5 µm) counter electrodes as shown in Fig. 2.

**Fig. 3:**
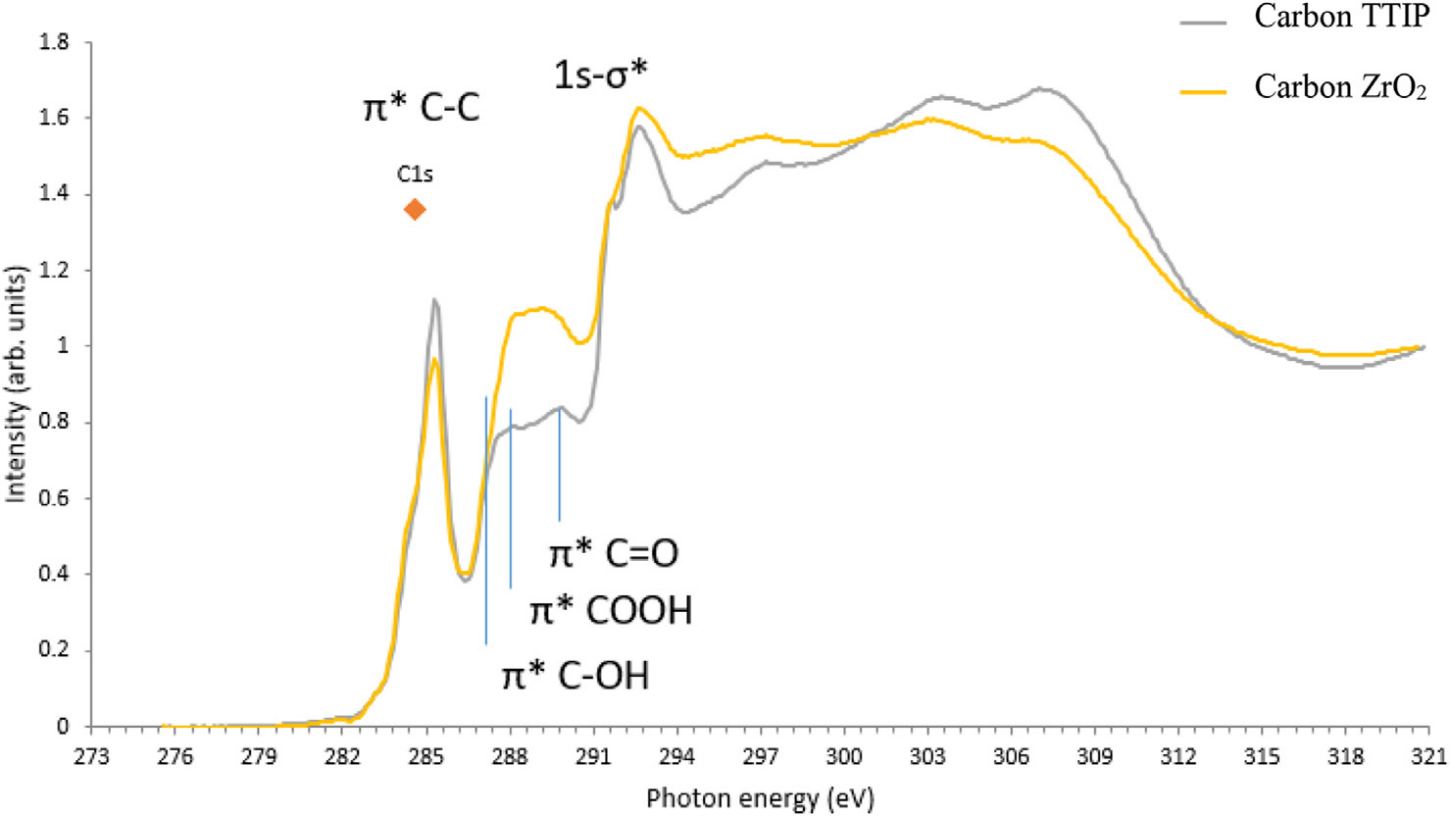
Sample data from the NEXAFS plots for CB/Gr:TTIP and CB/Gr:ZrO_2._ This plot was obtained from Synchrotron Light Research Institute, Thailand. The NEXAFS data spreadsheet can be found in the Supplementary data.

Meanwhile, the third level data is the near-edge X-ray absorption fine structure, NEXAFS. This data allows the exploration of the unoccupied electronic levels of a species which lie below or at the vacuum level. C K edges as shown in Fig. 3, which indicates the presence of excitonic states of 1s-π* and σ* in all spectra. C K edge may involve interlayer states such as π* C-O and C=O.

## 2 Experimental Design, Materials and Methods

Different amount of TTIP and ZrO_2_ binders were added to the carbon black-graphite paste. Each binder also has a different particle size. TTIP binder which formed TiO_2_ has a particle of ∼100 nm, while ZrO_2_ binders have a particle sizes of 0.22 µm and 5 µm, respectively. Each of the binder is added to the carbon black-graphite paste and was readily mixed until the paste become homogenous. The counter electrode was fabricated on FTO substrate by doctor blade method and sintered at 400 °C for 1 h. In the four-point probe, the needles of equal spacing between each of the probes were placed on the surface of the counter electrode. Current is then applied on the outer two probes, and voltage across the inner two probes is measured. The SEM procedure were carried out using back scattered electron. The films were cut 1 cm by 1cm. Meanwhile, NEXAFS were performed at 20° from the surface normal to analyze the binding energy of the elements. All spectra were measured at a photon energy of 600 eV, and excited photoelectrons detected in unit of counts per second were normalized with the incoming soft X-ray intensity, monitored by the drain current from the gold mesh in front of the sample. All experiments are conducted at room temperature.

## CRediT authorship contribution statement

**Nurnajaa Narudin:** Investigation, Writing – original draft. **Piyasiri Ekanayake:** Supervision. **Ying Woan Soon:** Writing – review & editing. **Hideki Nakajima:** Data curation, Formal analysis.

## Declaration of Competing Interest

The authors declare that they have no known competing financial interests or personal relationships which have, or could be perceived to have, influenced the work reported in this article.

## Appendix Supplementary Materials

Image, application 1

Image, application 2

Image, application 3
