# Supplementary figures and images for: Experimental data of four-point probe, scanning electron microscopy, and near-edge X-ray fine structure of titanium (IV) isopropoxide and zirconium (IV) dioxide binders incorporated carbon-based counter electrode for dye-sensitized solar cells
Source: Data Brief. 2021 Oct 17;39:107487. doi: 10.1016/j.dib.2021.107487 (PMC8554458; doi:10.1016/j.dib.2021.107487)

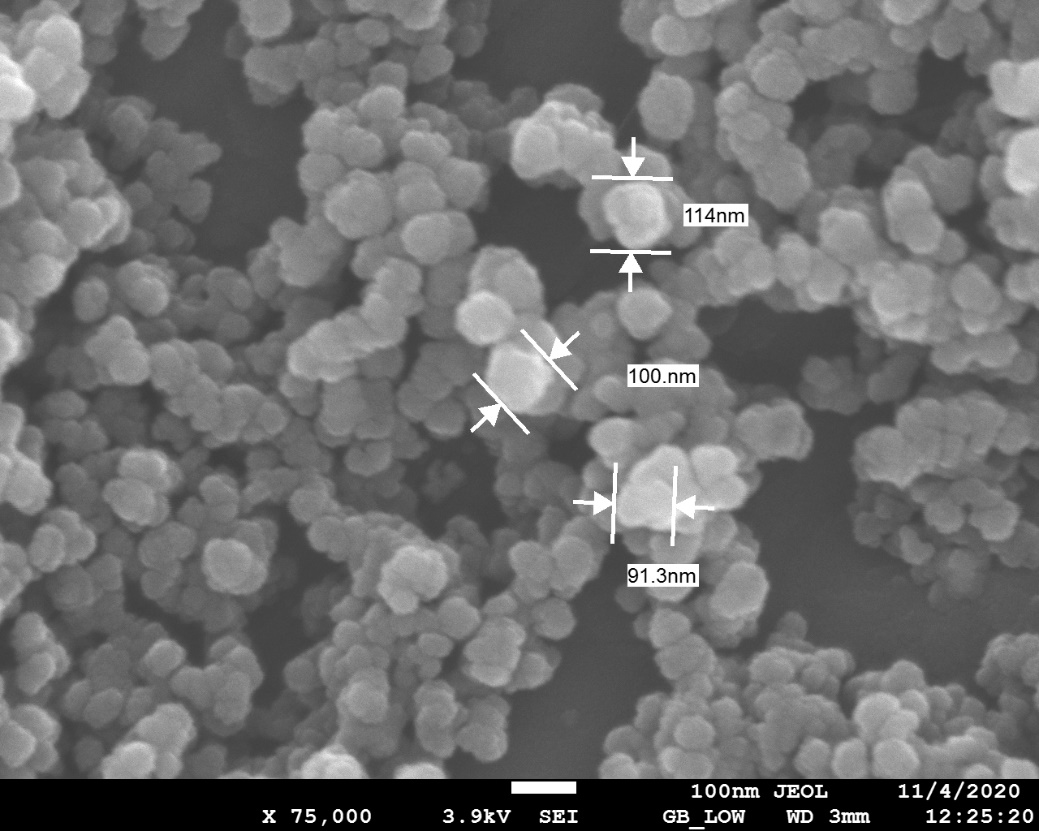


**SEM Images of ZrO2**


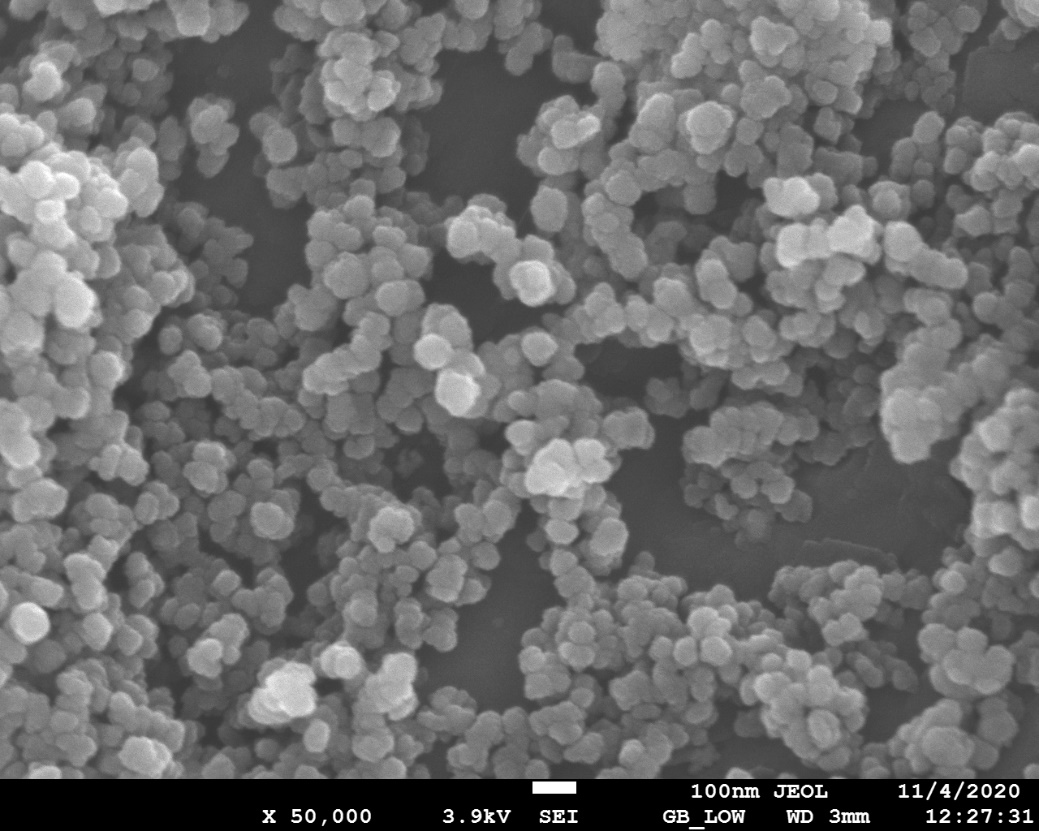


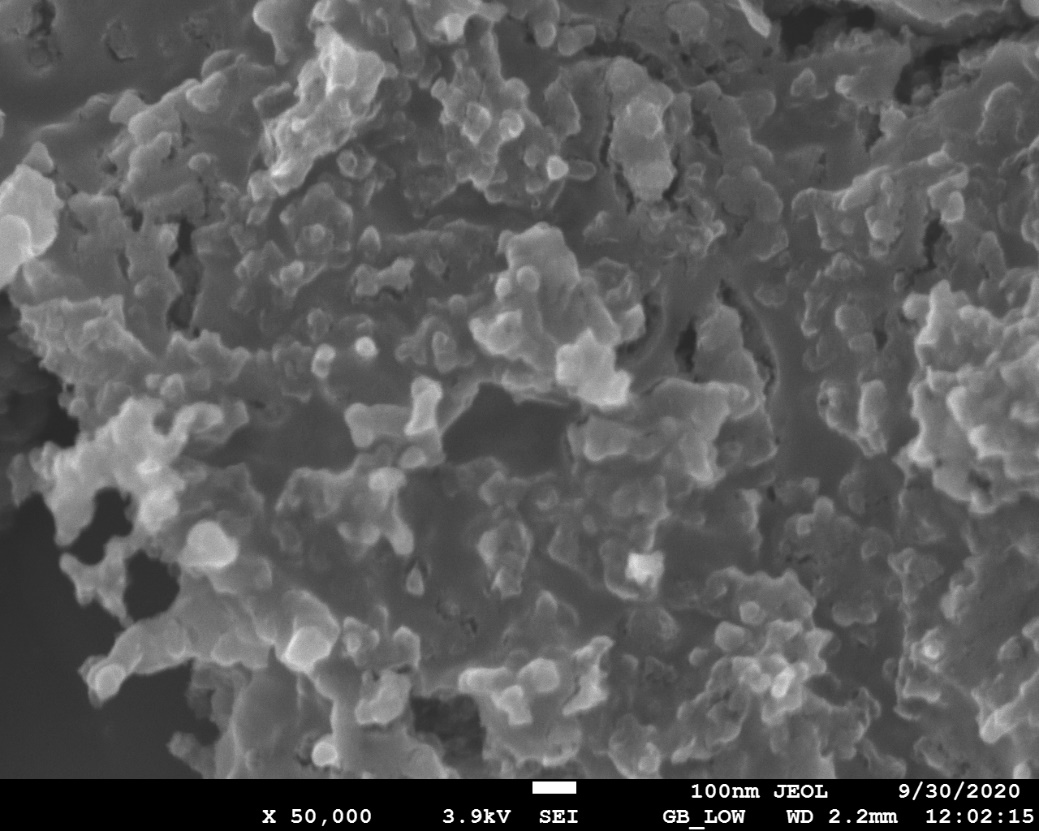

Supplement: Supplementary file 2 [file mmc2.docx]
