# Supplementary material for: Experimental data of four-point probe, scanning electron microscopy, and near-edge X-ray fine structure of titanium (IV) isopropoxide and zirconium (IV) dioxide binders incorporated carbon-based counter electrode for dye-sensitized solar cells
Source: Data Brief. 2021 Oct 17;39:107487. doi: 10.1016/j.dib.2021.107487 (PMC8554458; doi:10.1016/j.dib.2021.107487)

Project Notes

Cross sectional SEM images and EDX

Specimen Notes

CB/Gr:TTIP


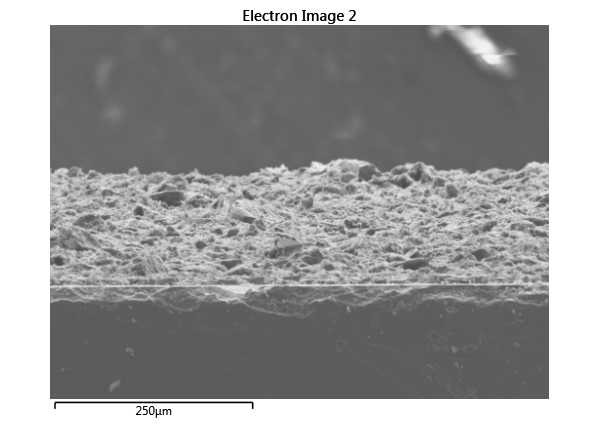


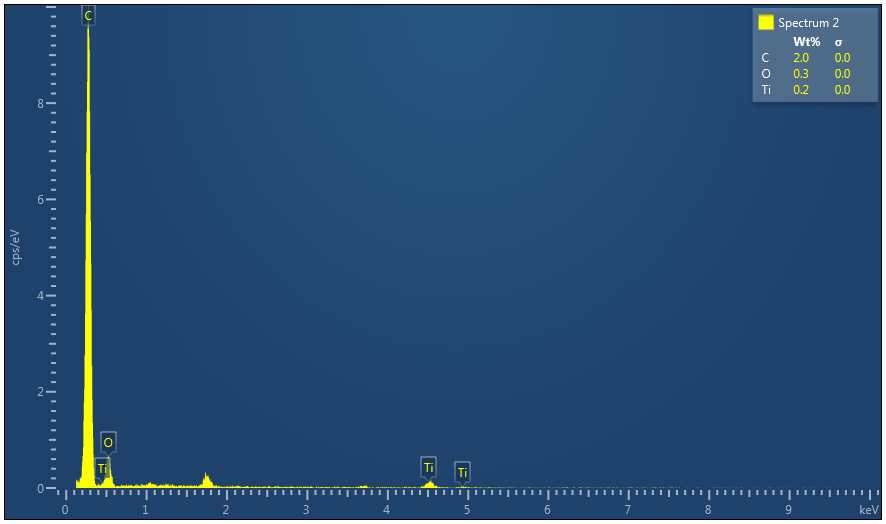


| Element | Line Type | Apparent Concentration | Intensity Correction | k Ratio | Wt% | Wt% Sigma | Atomic % | Standard Label | Factory Standard |
| --- | --- | --- | --- | --- | --- | --- | --- | --- | --- |
| C | K series | 1.74 | 0.89 | 0.01735 | 1.96 | 0.02 | 88.93 | C Vit | Yes |
| O | K series | 0.20 | 0.75 | 0.00068 | 0.27 | 0.02 | 9.23 | SiO2 | Yes |
| Ti | K series | 0.12 | 0.74 | 0.00120 | 0.16 | 0.01 | 1.84 | Ti | Yes |
| Total: |  |  |  |  | 2.39 |  | 100.00 |  |  |


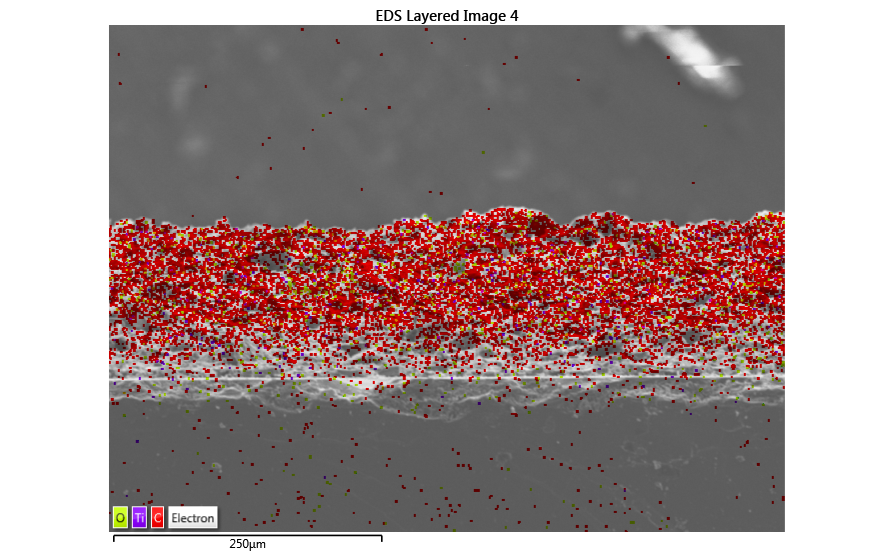


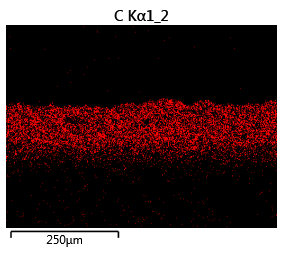

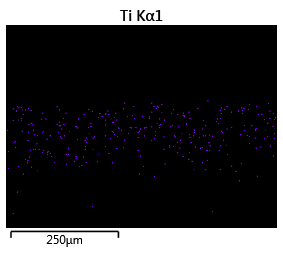

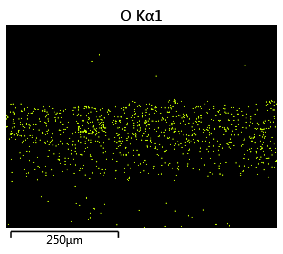


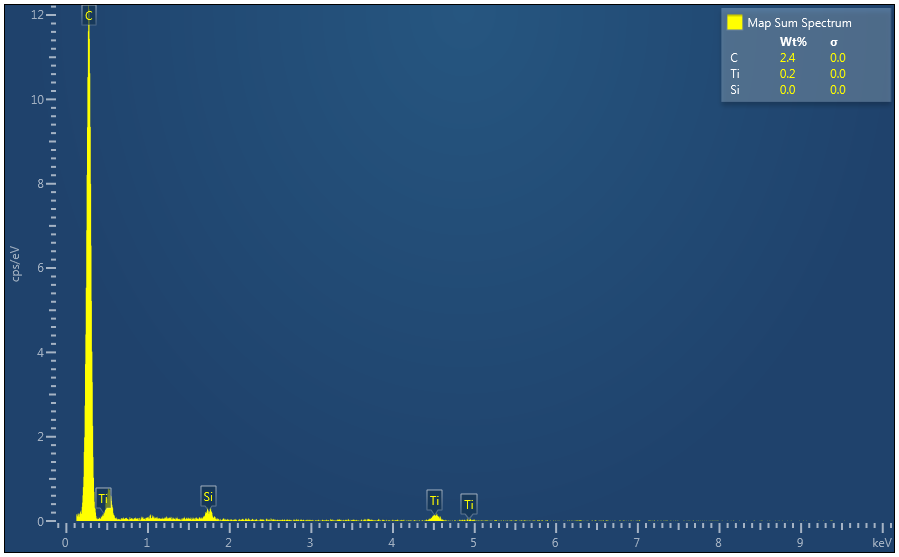

Supplement: Supplementary file 3 [file mmc3.docx]
